# PARP-14 Binds Specific DNA Sequences to Promote Th2 Cell Gene Expression

**DOI:** 10.1371/journal.pone.0083127

**Published:** 2013-12-20

**Authors:** Jonathan P. Riley, Aishwarya Kulkarni, Purvi Mehrotra, Byunghee Koh, Narayanan B. Perumal, Mark H. Kaplan, Shreevrat Goenka

**Affiliations:** 1 Department of Pediatrics, HB Wells Center for Pediatric Research, Indiana University School of Medicine, Indianapolis, Indiana, United States of America; 2 Department of Microbiology and Immunology, Indiana University School of Medicine, Indianapolis, Indiana, United States of America; 3 School of Informatics, Indiana University-Purdue University, Indianapolis, Indianapolis, Indiana, United States of America; National Cancer Institute (INCA), Brazil

## Abstract

PARP-14, a member of the poly ADP-ribose polymerase super family, promotes T helper cell 2 (Th2) differentiation by regulating interleukin-4 (IL-4) and STAT6-dependent transcription. Yet, whether PARP-14 globally impacts gene regulation has not been determined. In this report, using an RNA pol II ChIP-seq approach, we identify genes in Th2 cells that are regulated by PARP-14, and either dependent or independent of ADP-ribosyltransferase catalytic activity. Our data demonstrate that PARP-14 enhances the expression of Th2 genes as it represses the expression of Th1-associated genes. Among the relevant targets are Signal Transducer and Activator of Transcription genes required for polarizing Th1 and Th2 cells. To define a mechanism for PARP-14 function, we use an informatics approach to identify putative PARP-14 DNA binding sites. Two putative PARP-14 binding motifs are identified in multiple Th2 cytokine genes, and we demonstrate that PARP-14 interacts with each motif using in vitro binding assays. Taken together our results indicate that PARP-14 is an important factor for T helper cell differentiation and it binds to specific DNA sequences to mediate its function.

## Introduction

The cytokine interleukin-4 (IL-4) activates the Signal Transducer and Activator of Transcription 6 (STAT6) to mediate its function [1], [2], [3], [4]. Receptor engagement by IL-4 leads to Janus kinase-mediated tyrosine phosphorylation of latent STAT6. After tyrosine phosphorylation, STAT6 forms dimers, translocates to the nucleus, and binds to specific DNA sequences to regulate gene transcription. The DNA binding sites for STAT6 consist of palindromic sequences (TTCN_3–4_GAA) with an N3–N4 spacer between the inverted repeats [5], [6]. Both IL-4 and STAT6 play an important role in T helper cell immune responses, specifically in the type 2 response (Th2) [2], [4], [7]. The Th2 responses are associated with humoral immunity and provide help for antibody dependent immune responses [2], [4], [5], [7]. Th2 immune responses are typically elicited against extracellular parasites including helminthes [4], [5]. Moreover, dysregulated Th2 immune responses are associated with allergic disorders including asthma, atopic dermatitis and food allergies [8], [9], [10], [11], [12], [13], [14].

Previously, we have identified PARP-14 (poly ADP ribose polymerase) as a factor that specifically interacts with STAT6 to induce the expression of IL-4-dependent genes [15], [16], [17]. Several conserved domains are found in PARP-14 including, three copies of the macro domain and a PARP catalytic domain [15]. The macro domains were first identified in the non-classical histone macroH2A (mH2A) [18]. The PARP domain found in PARP-14 was first identified in PARP-1 [19], and 16 additional proteins have been identified that contain the PARP catalytic domain and collectively form the PARP super-family of proteins [20]. Recently, this family of proteins has been defined using an alternate nomenclature and are called ARTDs (ADP-ribosyltransferase diphtheria toxin-like), with PARP-14 (standard gene symbol *Parp14*) also known as ARTD8 [21]. The PARP catalytic domain contains an enzymatic activity that uses NAD as a substrate and transfers ADP-ribose moieties on protein acceptors, including itself. The quintessential function of PARP-1, the most characterized protein of this family, is in DNA damage repair and in the manifestation of an inflammatory response due to oxidative stress [19], [20]. Due to its central role in two important cellular processes considerable effort has been spent on developing pharmacological inhibitors that interfere with the poly(ADP-ribosyl)ation activity of PARP-1 [22]. Most PARP inhibitors act as competitive inhibitors as they occupy the NAD binding site within the catalytic domain of the enzyme [22]. Water soluble PARP inhibitors, including N-(6-oxo-5,6-dihydro-phenanthridin-2-yl)-N, N-dimethylacetamide HCl (PJ34), are available and have been used in vivo to exert anti-inflammatory actions [23]. As PJ34 is a mimic of NAD it is not specific for an individual PARP enzyme and has been shown to inhibit a number of PARP family members including PARP-14 [24].

PARP-14 interacts with STAT6 and enhances its transcription activity. Our data demonstrated that PARP-14 functions as a transcriptional switch for STAT6 dependent gene induction. In the absence of IL-4, PARP-14 was found to be bound to STAT6 responsive promoters, and functioned as a transcriptional repressor by recruiting HDAC 2 and 3. In the presence of IL-4 the catalytic activity of PARP-14 modified the HDACs and the repressive complex was displaced from the promoter to activate transcription [25]. PARP-14 is required for STAT6-dependent gene expression in B cells and T helper cells [24], [25], [26]. These data indicated that PARP-14 has the ability to bind DNA but the exact sequence to which PARP-14 binds is not known. Moreover, whether PARP-14 functions only with STAT6 in Th2 cells, or if it has STAT6-independent functions, is not known.

To investigate the role of PARP-14 in Th2 cells we performed a high throughput sequencing study to define the active gene transcription in Th2 cells that was dependent on PARP-14 and/or ADP-ribosyltransferase (ART) activity. T helper cells from *Parp14*+/+ and *Parp14*−/− were differentiated under Th2 conditions with and without PJ34, and were used for ChIP-Seq analysis to identify genes that were actively transcribed by using an antibody directed against the active form of RNA polymerase II. Actively transcribed genes identified from this analysis were divided based on a requirement for PARP-14 and PARP enzyme activity. We used genomic sequences from the genes that were positively regulated by PARP-14 and performed a de novo search for common sequences and identified putative binding sites for PARP-14 present in Th2 cytokine loci. Thus, PARP-14 binds to specific DNA sequences in regulating the expression of genes in Th2 cells.

## Materials and Methods

### Ethics Statement

All animal experiments were approved by Indiana University School of Medicine Institutional Animal Care and Use Committee.

### Mice

Six to eight week old C57BL/6 and *Parp14*−/− mice on a C57BL/6 background were used for all studies. Mice were maintained in pathogen free conditions.

### ChIP-Seq

Spleens were harvested from 6–8 week old C57BL/6 wild-type and *Parp14*−/− mice. Naïve CD4+ T cells were isolated with a CD4^+^ CD62L^high^ T cell Kit and MACS separation columns (Miltenyi Biotec, Auburn, CA) as described by the manufacturer. Naïve CD4^+^ T cells were cultured in RPMI as previously described [6] in the presence or absence of 5 µM PJ34 as indicated. Cells were then fixed according to the Active Motif (Carlsbad, CA) cell fixation protocol for ChIP assays. ChIP-Seq was performed by Active Motif using the TranscriptionPath service. Briefly, chromatin was immunoprecipitated using an antibody specific for RNA Polymerase II phosphoserine 2. To verify the quality of the ChIP DNA, a TranscriptionPath qPCR assay was carried out using primers that amplify a genomic region in the 1st introns of the *Actb* (Actin B) gene and the Th2-induced *Il5* gene. Quantitative PCR was performed again following amplification of the ChIP DNA required to generate adequate DNA for sequencing. 36-nt sequence reads were identified by the Sequencing Service (using Illumina’s Genome Analyzer 2). At least 28 million quality-filtered reads per sample were mapped to the mouse genome (mm9) using the ELAND (Illumina) algorithm. After removal of duplicate reads, the data files for the 4 samples and Input were normalized to 8.2 million uniquely-mapped alignments each. SICER analysis [27] was then performed to identify peaks of enriched RNA Polymerase II protein binding (range of 8840–12014/sample) in samples compared to the input file. SICER analysis was performed with the following parameters: Species-mm9; redundancy threshold-1; window size-150 bp; fragment size-150 bp; effective genome fraction-0.86; gap size-450 bp; FDR-10^−10^]. ChIP-seq data files were submitted to GEO (accession number GSE51344).

### Gene Expression Analysis

Total RNA was purified using the TRIzol reagent (Invitrogen). cDNA was prepared using the SuperScript First-Strand cDNA synthesis system (Invitrogen). Quantitative RT-PCR (qRT-PCR) was performed for the indicated genes using the comparative threshold cycle method and normalized to *b2m*.

### Immunoblot Analysis

Whole cell protein lysates extracted from *in vitro* differentiated cells were imunoblotted with anti-RNA polymerase II CTD phospho-Serine 2 (ActiveMotif) and RNA polymerase II (ActiveMotif) as a control.

### Homer Analysis for Determination of the DNA Binding Site for PARP-14

HOMER was used to perform a de novo motif analysis using the findMotifs.pl tool [28]. HOMER uses ZOOPS scoring (zero or one occurrence per sequence) coupled with the hypergeometric enrichment calculations (or binomial) to determine motif enrichment. Genes that were positively regulated by PARP-14 were used as the list of target genes, and genes that showed no regulation by PARP-14 were used as the list of background genes. The region of each gene from 1000 base pairs upstream to 100 base pairs downstream of the transcription start site for each gene was used in the analysis.

### DNA Affinity Pull-Down Assay

Double-stranded biotinylated oligonucleotides (Il4-GCCAAGCTTGTGAGTCTGAG**TTCAAGGATC**CACACGGTGCAAAGAGAGAC, Il4 scramble-GCCAAGCTTGTGAGTCTGAG**CGTGCATGCA**CACACGGTGCAAAGAGAGAC, Il5-TTACTAAAAGGCCAACCCA**GACTGAGTGGAG**ATAAGAGGATGCTTCTTGG, Il5 scramble-TTACTAAAAGGCCAACCCA**GCAGATTGCTTT**ATAAGAGGATGCTTCTTGG, and the respective reverse complements) were incubated with streptavidin-agarose beads (Millipore, Billerica, MA) for thirty minutes at 4°C. The beads were then washed with pull-down buffer to remove unbound oligonucleotides. The oligonucleotide-bead complexes were then incubated with 100 µg of salmon sperm DNA and 500 µg of protein extract, from 293T cells transfected with PARP-14 cDNA. The reaction was carried out for 2 hours in pull-down buffer composed of 25 mM HEPES, 15 mM NaCL, 0.5 mM DTT, 0.1 mM EDTA, 10% glycerol, and 0.5% Nonidet P-40 at 4°C. The beads were then washed with pull-down buffer to remove nonspecific protein binding. The proteins still bound to the oligonucleotides were then identified by Western blotting. Quantification of protein bands was determined using ImageJ [29].

### Dual Luciferase Assay

The CMV PARP-14 expression vector [25] (0.5 µg/ml), and pGL3-IL5P reporter vector [30] (0.5 µg/ml) and *Renilla* luciferase reporter plasmid (125 ng/ml) as an internal control were transfected into Jurkat T cells using FuGene6. After 24 hours, cells were stimulated with PMA (50 ng/ml) and ionomycin (500 ng/ml) for 6 hours before isolation of cell extracts. Luciferase activity was measured by using the Dual-Luciferase Reporter Assay System (Promega) according to the manufacturer’s instructions.

## Results

### Determination of Genome Wide Transcription in Th2 cells Dependent on PARP-14 and ADP-ribosyltransferase Activity

**Figure 1:**
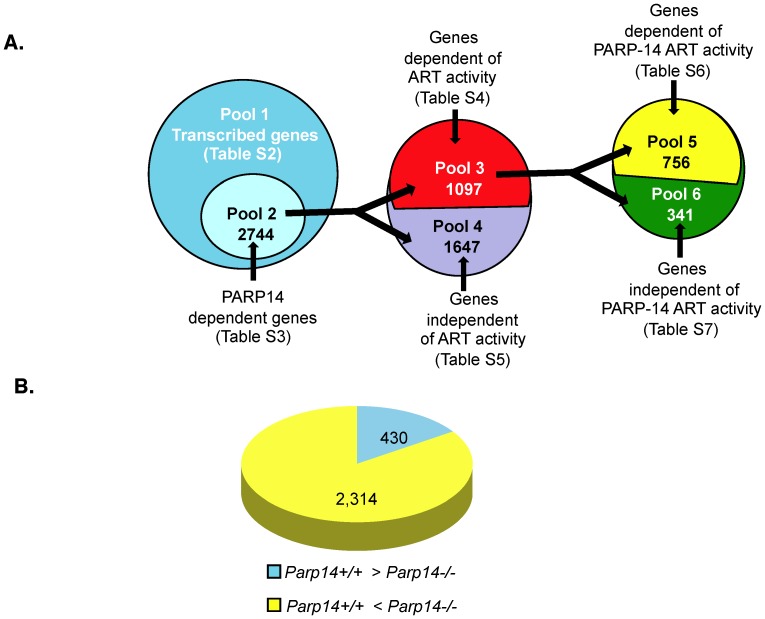
PARP-14 and its enzyme activity regulate expression of a broad range of genes. (A) Graphical illustration of numerical distribution of genes determined by RNA Poymerase II ChIP-seq in Th2 cells that are regulated by PARP-14. Pool 1 represents genes undergoing significant transcription as determined by ChIP-Seq. Pool 2 includes genes that are dependent on the expression of PARP-14. Pool 4 contains genes that are independent of the ART activity of PARP-14. Pool 3 contains genes dependent on PARP-14 and ART activity, and was further divided into Pool 5, genes that are dependent on the ART activity of PARP-14 and Pool 6 containing genes that depend on ART activity of PARP-14 and other PARP enzymes. (B) Graphical representation showing proportion of genes positively regulated by PARP-14 (yellow) and genes negatively regulated by PARP-14 (blue).

To determine genes that are regulated by PARP-14 and ADP-ribosyltransferase (ART) activity at the genomic level, we performed RNA Polymerase II ChIP-Seq analysis on *in vitro* differentiated Th2 cells from *Parp14+/+* and *Parp14−/−* mice that were cultured in the absence or presence of the ART inhibitor PJ34. The RNA Polymerase II ChIP-Seq showed a total of 25,677 genes with active gene transcription in at least one of the four Th2 cell samples (Table S1). Active transcription was ascertained based on average peak values of sequence tags associated within a gene. In order to eliminate genes that did not have significant active gene transcription, all genes that did not have at least one sample with an average peak value above 0.500, or an average peak value above the input sample were removed from further analysis. The remaining genes were cross-referenced with the list of genes analyzed by SICER, and genes that did not have a significant level of active gene transcription in at least one sample were removed from further analysis. A total of 8,062 genes were determined to have active transcription occurring in at least one sample. All subsequent analyses utilized this revised list of 8,062 genes (Pool 1) (Figure 1A and Table S2).

In order to determine the genes whose expression is dependent on PARP-14, we compared the average peak values of the ChIP-Seq signal in wild-type and PARP-14 deficient samples. A gene was determined to be down-regulated in the absence of PARP-14 if the average peak value ratio between the *Parp14+/+* and *Parp14−/−* sample was greater than 1.4. Similarly, gene expression was defined as increased in the *Parp14−/−* compared to the wild-type sample if the ratio was less than 0.7. Although these ratios are effective for most genes, genes that are actively transcribed at significantly higher levels (an average peak value greater than 2) may show a less significant *Parp14+/+* to *Parp14−/−* ratio, and might be eliminated as a false negative. To correct for this, all genes that had a *Parp14+/+* to *Parp14−/−* ratio between 1.2–1.4 and 0.7–0.83 were included only if they had an average peak value above 2 in either of the samples. To ensure that the genes on this list showed significant transcription, the sample with the higher signal was verified to be a significant peak by SICER analysis. With this analysis a list of 2,744 genes were then identified as genes dependent on PARP-14 (Pool 2) (Figure 1A, Table S3). Amongst these 2,744 genes 2,314 genes were positively regulated by PARP-14 as the active transcription was higher in *Parp14*+/+ as compared to *Parp14*−/− samples, and 430 genes showed a higher level of transcription in *Parp14*−/− than *Parp14*+/+ indicating a repressive role of PARP-14 in transcription (Figure 1B).

To determine the genes whose transcription is dependent on the ART activity of only PARP-14, we began further analysis from Pool 2. We reasoned that elimination of PARP-14 would cause a difference in active transcription that was either dependent or independent of the enzyme activity of PARP-14. In order to determine whether a gene is affected by the ART activity of PARP-14, PARP-14 would need to be present to ensure that treatment with a PARP inhibitor (PJ34) would have an effect on the PARP activity specific to PARP-14. Therefore, we took genes in Pool 2 and compared the wild-type sample with the *Parp14*+/+ + PJ34 sample for each gene. If there was no significant difference in levels of active gene transcription between the untreated wild-type sample compared to wild-type+PJ34 sample (a ratio of 0.83–1.2 between WT and WT+PJ34), then the gene was classified as independent of ART activity. Genes regulated by PARP-14 and ART activity were designated as Pool 3 (1,097 genes) (Figure 1A and Table S4). We further analyzed Pool 3 to identify genes whose transcription was dependent on the ART activity of only PARP-14, or was dependent on the ART activity of PARP-14 and/or other PARPs. Genes in Pool 3 that had no significant difference between the *Parp14−/−* and *Parp14−/−* + PJ34 samples (ratio of 0.83–1.2) were genes that were regulated by the ART activity of only PARP-14 (756 genes in Pool 5) (Figure 1A and Table S6). The remaining 341 genes were regulated by the ART activity of other PARP enzymes (Pool 6) (Figure 1A and Table S7).

To determine the genes whose transcription regulation is independent of the ART activity contained in PARP-14, we started our analysis with the genes that had a significant difference between the wild-type and *Parp14−/−* samples (Pool 2). We narrowed down the list by including genes that showed no significant difference between the untreated and PJ34-treated samples for *Parp14+/+* (ratio of 0.83−1.2). Thus, this list consists of 1,647 genes that are affected by the loss of PARP-14 but are not affected by the inhibition of the ART activity of PARP-14 (Pool 4) (Figure 1A and Table S5).

### PARP-14 and ART Activity Regulate Expression of Genes Associated with a Number of Cellular Pathways

**Figure 2:**
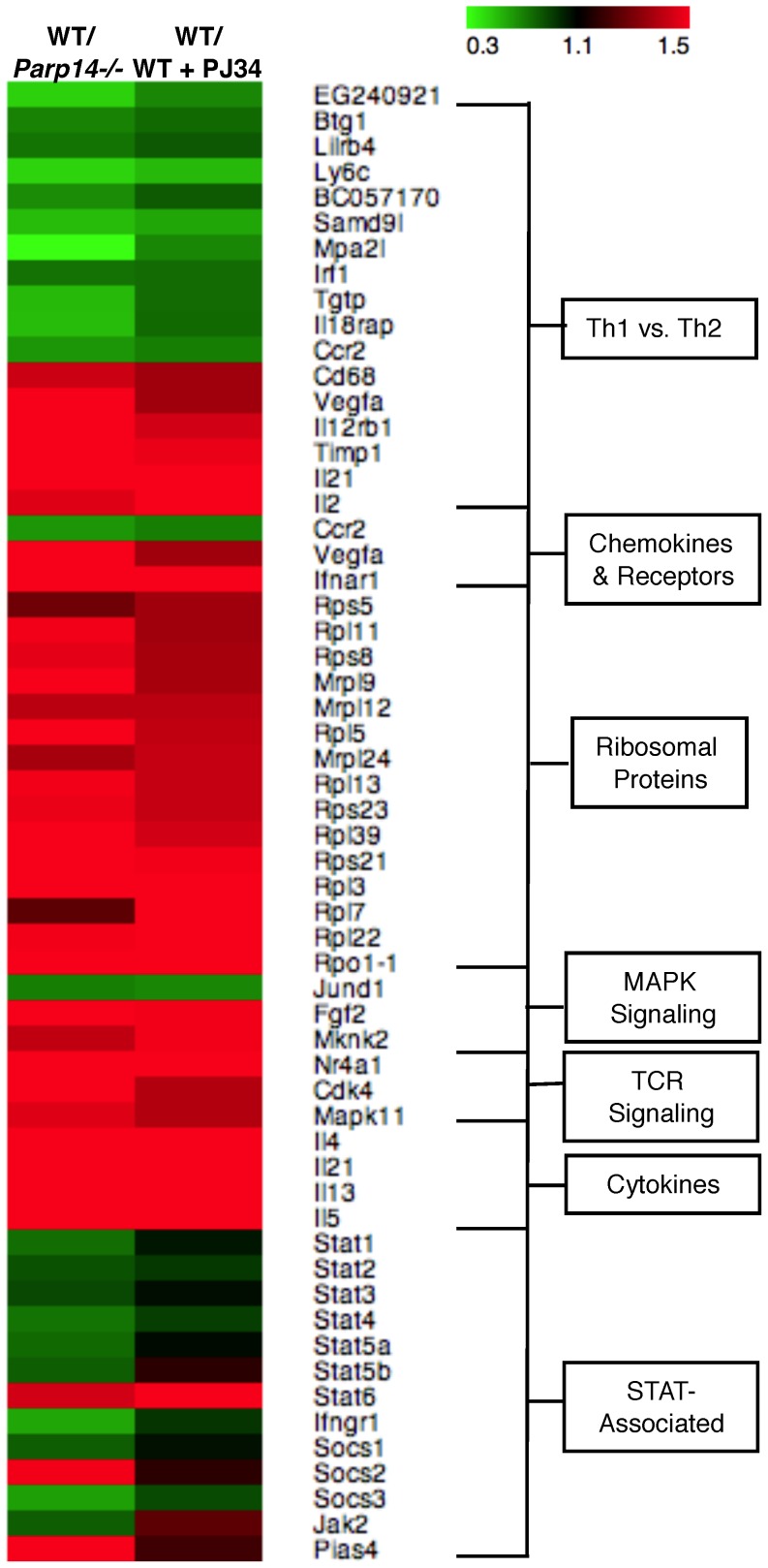
PARP-14 regulates genes that participate in diverse cellular pathways. Heat map indicating the level of dependence on PARP-14 for expression of the indicated genes. Rows represent individual genes involved in the indicated cellular pathways. The data in column 1 represent the comparison of wild type versus Parp14−/− Th2 cells, and data in column 2 represent the comparison of wild type Th2 cells cultured in the presence or absence of PJ34. Green and red indicate negative and positive regulation respectively.

To classify the genes regulated by PARP-14 into functional categories we used the Database for Annotation, Visualization and Integrated Discovery (DAVID) tool [31], [32]. Using this algorithm we were able to determine that the genes regulated by PARP-14 were involved in a number of cellular processes (Table 1). PARP-14 regulated a large number of genes involved with the ribosomal machinery, and other functional pathways including T cell receptor signaling, ubiquitin mediate proteolysis, cell cycle, MAPK signaling, oxidative phosphorylation, and the JAK-STAT pathway (Table 1). To further determine whether PARP-14 and ART activity were regulating the expression of genes found in these cellular pathways, we constructed a heat map for a subset of these genes based on the ratio of gene expression in wild type versus *Parp14*−/− Th2 cells (column 1) or wild type Th2 cells treated or untreated with PJ34 (column 2) (Figure 2). We observed that *Irf1*, *Stat1* and *Stat4* involved in Th1 differentiation [33], [34], [35], [36] were negatively regulated by PARP-14 and ART enzyme activity (Figure 2). Conversely, Th2 specific genes including *Il4*, *Il5* and *Il13* were positively regulated by PARP-14 and ART enzyme activity. A large number of genes associated with the ribosomal machinery were positively regulated by PARP-14 and ART enzyme activity (Figure 2). Taken together, these data suggest that PARP-14 appears to play a significant role in regulating a broad set of cellular functions including T-cell associated functions such as Th1/Th2 polarization, T-cell receptor signaling, and cytokine signaling.

**Table 1:** PARP-14 regulates genes involved in a number of cellular pathways as determined by the DAVID tool.

Genes Regulated by PARP-14					
Pathway Category	Pathway	# Genes	Percent Total	P-Value	Benjamini
KEGG	Ribosome	59	2.6	6.9E-31	1.2E-28
KEGG	T Cell receptor signaling pathway	39	1.7	3.3E-08	3.0E-06
KEGG	JAK-STAT signaling pathway	43	1.9	7.8E-07	4.6E-05
KEGG	Ubiquitin Mediated proteolysis	38	1.7	5.3E-06	2.4E-04
KEGG	Cell cycle	34	1.5	5.6E-05	1.7E-03
KEGG	MAPK signaling pathway	56	2.5	1.9E-04	4.1E-03
KEGG	Oxidative phosphorylation	27	1.2	1.4E-02	1.3E-01

Results from the DAVID tool using the KEGG (Kyoto Encyclopedia of Genes and Genomes) sourced pathways on genes that are regulated by PARP-14 (Pool 2). Number of genes regulated by PARP-14 in each of the listed pathways is indicated, and the percent total indicates the percentage of genes within a pathway that are regulated by PARP-14, with p-values and Benjamini values indicating the chance of false positive identification.

### Transcriptional Regulation of STATs and STAT-associated Genes by PARP-14

**Figure 3:**
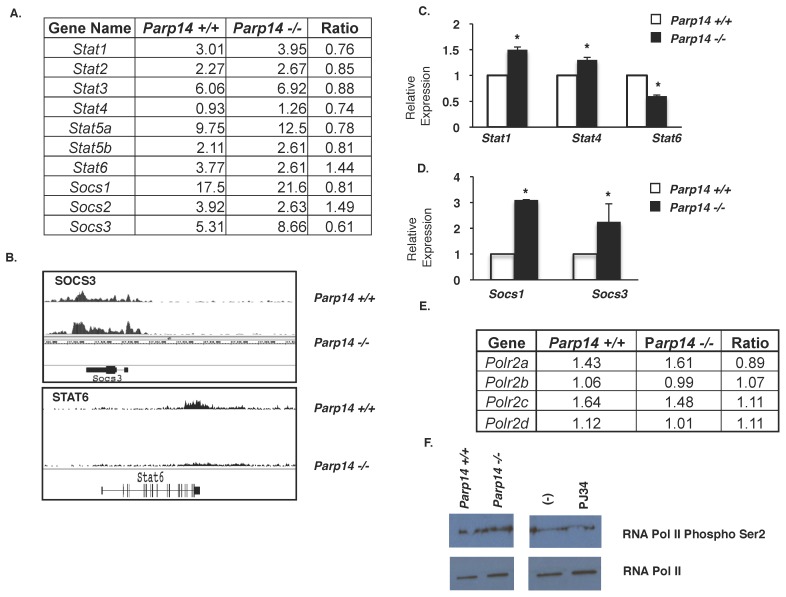
PARP-14 regulates expression of some of the STAT factors and STAT-associated genes. (A) Values in table represent the average peak values of RNA Polymerase binding for each of the indicated genes in both *Parp14*+/+ and *Parp14*−/− samples. (B) Integrated Genome Browser figures of RNA pol II binding to the *Socs3* and *Stat6* genes in wild type and *Parp14*−/− T cells. (C–D) Splenic CD4 T cells were isolated from *Parp14*+/+ and *Parp14*−/− mice. Total RNA was isolated and transcript levels for the indicated STAT factors (C), and SOCS factors (D) were quantified using quantitative PCR. Values plotted are means ± SEM from three independent experiments. (E) The table shows the average peaks of RNA polymerase II isoforms, which are actively transcribed. (F) Naïve CD4 T cells isolated from *Parp14+/+* mice and *Parp14−/−* were cultured under Th2 conditions, with or without PJ34 as indicated, for 7 days and restimulated with IL-4 for 2 hours. Total extracts were immunoblotted for RNA polymerase II phospho-Ser2 and total RNA polymerase II as control.

One of the pathways identified by the DAVID analysis and impacted by PARP-14 was the JAK-STAT pathway, and specifically STAT proteins themselves (Figure 3). The Th1-associated *Stat1* and *Stat4* genes were negatively regulated by PARP-14 and the Th2-associated *Stat6* gene was positively regulated by PARP-14 as seen in gene expression ratios and Integrated Genome Browser figures (Figure 3A–B). To confirm this ChIP-Seq data we isolated splenic CD4+ T cells from PARP-14 deficient and wild-type mice and analyzed the expression of *Stat1*, *Stat4* and *Stat6*. In agreement with our ChIP-Seq data, the expression of *Stat1* and *Stat4* was higher in *Parp14*−/− samples compared to wild-type, and the expression of *Stat6* was significantly lower in the *Parp14*−/− cells compared to *Parp14*+/+ cells (Figure 3C).

We further evaluated the expression of *Socs1*, *Socs2* and *Socs3*, known regulators of the STAT transcription factors [37], [38]. *Socs1* and *Socs3* were negatively regulated by PARP-14, and the converse was true for *Socs2* indicating a positive role of PARP-14 for its expression (Figure 3A–B). To confirm this data we measured transcript levels of *Socs1* and *Socs3* in differentiated Th2 cells by quantitative gene expression analysis. In agreement with the ChIP-Seq data, we observed that *Socs1* and *Socs3* expression was significantly increased in the absence of PARP-14 (Figure 3D).

As a control for the specificity of these observations, we also analyzed the expression of genes in RNA polymerase II family. Four family members showed modest changes in Pol II binding to the loci (Fig. 3E). The lack of a significant change in expression was confirmed by immunoblot analysis on protein extracts from *in* *vitro* differentiated wild type and *Parp14*−/− Th2 cells. We observed no defect in RNA Pol II expression or phosphorylation (Figure 3F). Similarly, PJ34 treatment had no effect on RNA pol II expression or phosphorylation (Figure 3F). These data indicate that PARP-14 regulates the expression of STAT transcription factors and STAT regulators including *Socs* genes.

### Analysis of Genes that are Regulated by STAT6 and PARP-14

**Figure 4:**
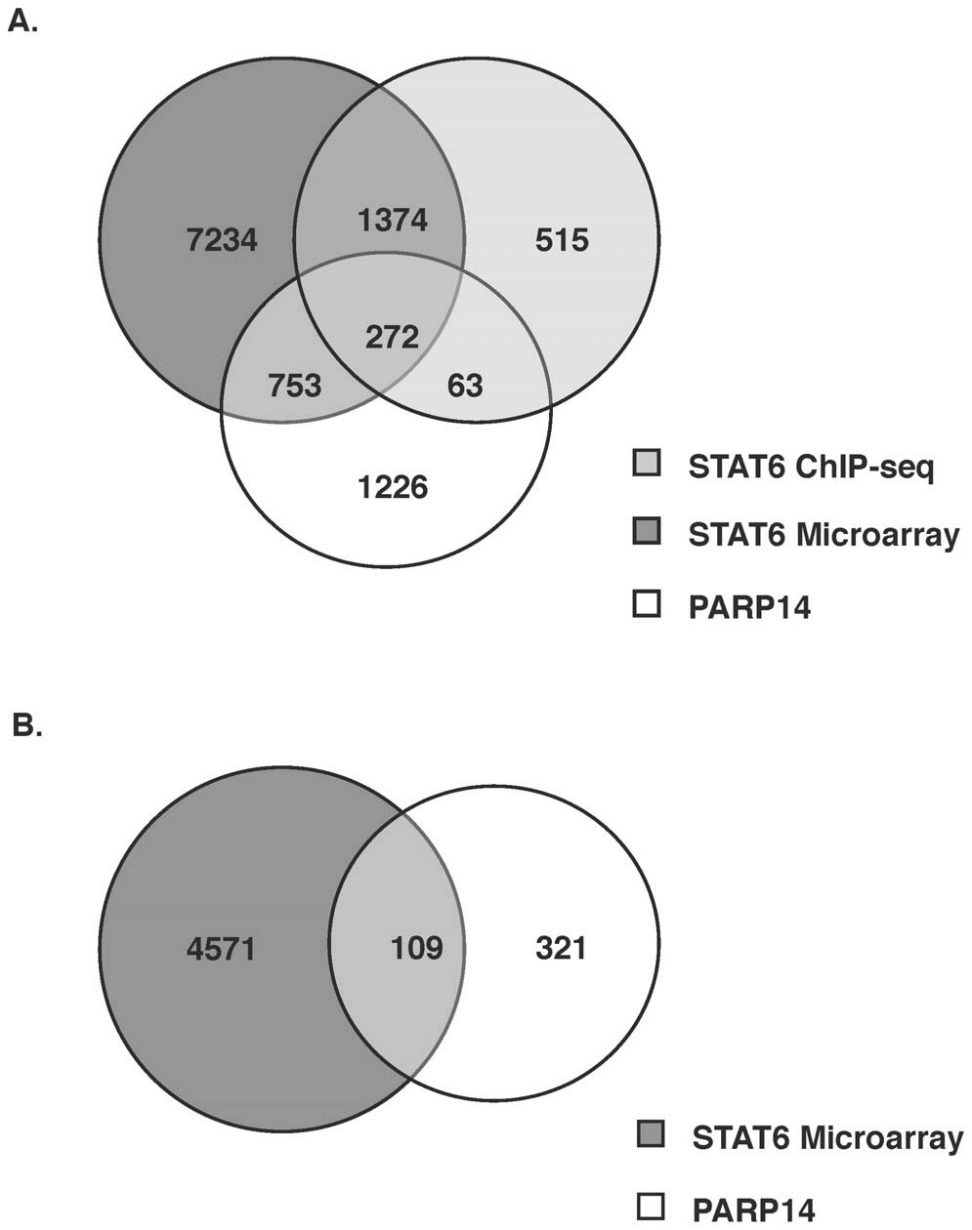
Comparison of genes that are regulated by STAT6 and PARP-14. (A) Genes identified by Wei et al that are positively regulated by STAT6 and bind STAT6 were compared to genes that are positively regulated by PARP-14 identified in Pool 2 of this study. (B) Genes identified by Wei et al that are negatively regulated by STAT6 were compared to genes that are negatively regulated by PARP-14.

PARP-14 had been initially identified as a cofactor that enhances STAT6 dependent transcription. Thus, to investigate on a genomic scale the identity of genes that are dependent on both STAT6 and PARP-14, we compared our PARP-14 data with the STAT6 data generated by Wei et al [6]. The culturing conditions were identical in both of these studies, which made this a valid comparison. The genes from the microarray data that were dependent on STAT6 were filtered using the same criteria as that used for identifying genes dependent on PARP-14. The genes on these lists were divided based on whether they were positively or negatively regulated by STAT6 and PARP-14 (Figure 4A and B respectively) and were compared. The positively regulated genes by both these factors were also compared to the genes identified by Wei et al as showing STAT6 binding by ChIP-Seq. There were 9,633 genes that showed positive regulation by STAT6, 2,314 genes were dependent on PARP-14 and 2,224 genes showed STAT6 binding. Our comparison yielded 272 genes that were positively regulated by both STAT6 and PARP-14 and also demonstrated STAT6 binding (Figure 4A, Table S8 Tab 1). Among the genes on this list were *Gata3*, *Il4* and *Il21*. As indicated in Figure 4A and Table S9, we found 753 genes that were independent of STAT6 binding but were positively regulated by both STAT6 and PARP-14. 1,374 genes showed positive regulation by STAT6 and binding of STAT6, but were not positively regulated by PARP-14. Only 63 genes showed STAT6 binding and were positively regulated by PARP-14 but not STAT6. When we compared gene lists that were negatively regulated by both factors, we found 109 common genes. Some of the genes on this list included *Stat1* and *Irf1*, genes involved in Th1 function (Figure 4B, Table S9). Although there is the limitation of this analysis that STAT6-dependent and PARP-14-dependent genes were identified through separate techniques, these results suggest that even though PARP-14 was identified as a STAT6 co-factor, each factor might have some separable functions. Thus, PARP-14 may function with additional transcription factors to regulate gene expression in Th2 cells.

### Identification of DNA Binding Motifs for PARP-14

**Figure 5:**
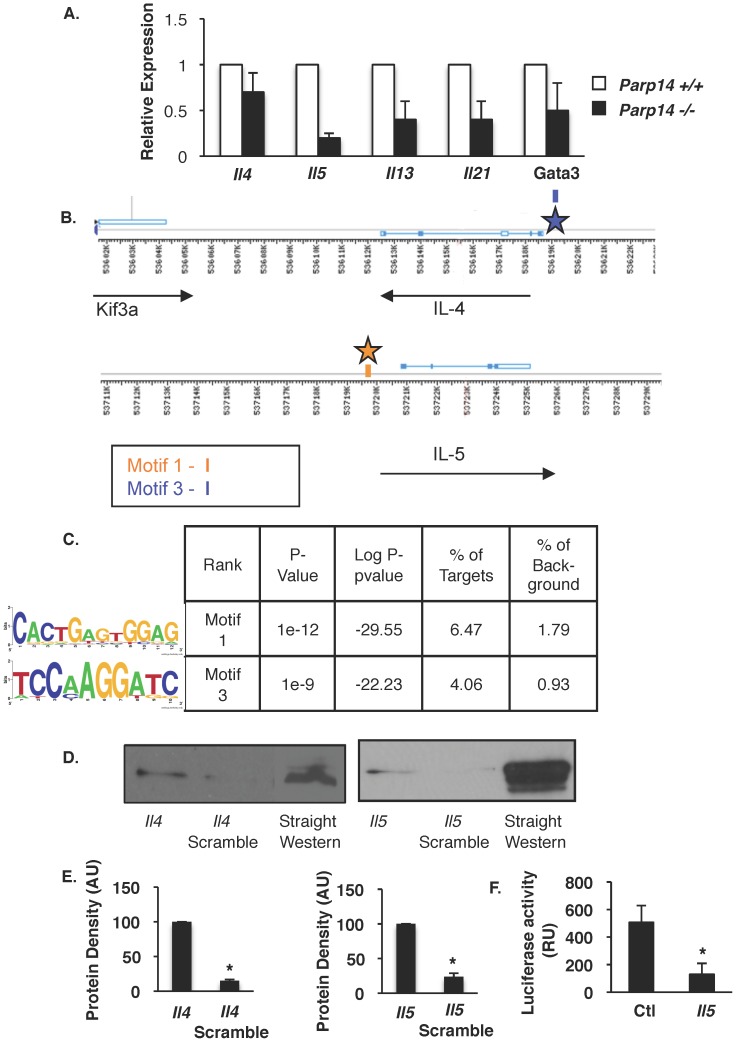
Putative DNA binding sites for PARP-14 identified by HOMER analysis, and PARP-14 binds to motif 3 and 1 found in the promoter region of *Il4* and *Il5* respectively. Naïve CD4 T cells were isolated from spleens of *Parp14*+/+ and *Parp14*−/− mice and cultured under Th2 conditions for 7d and were then restimulated for 2 hours with IL-4. (A) Total RNA was isolated and transcript levels for the indicated cytokines and transcription factor were quantified. Mean values ± SEM from three independent experiments are plotted. (B) Localization of putative PARP-14 binding sites on the *Il4* and *Il5* loci. This illustration depicts locations of putative PARP-14 binding sites determined by whole-gene HOMER analysis. Orange Bars represent occurrences of Motif 1. Blue bars represent occurrences of Motif 3. Stars indicate the regions used for DAPA experiments with PARP-14. (C) HOMER analysis used genes positively regulated by PARP-14 and searched for conserved binding motifs compared to a background list of genes that showed no regulation by PARP-14. Analysis was performed using 1000 bp upstream of gene transcription start site (TSS) and 100 bp downstream of TSS (1100 bp total/gene). Table shows P-value as likelihood of motif randomly occurring, Log P-value indicates enrichment, % of targets indicates the percentage of target genes in which the specified motif was found and % of background represents the percentage of background genes with the specific motif. (D) DNA Affinity Pull-down Assays (DAPA) were performed using lysates obtained from 293T cells transfected with a plasmid containing PARP-14 cDNA. The lysates were incubated with the indicated double stranded 50 bp oligonucleotides. Proteins bound to oligonucleotide were isolated and analyzed by Western blot using an antibody specific for PARP-14. (E) ImageJ was used to quantify signals from three independent experiments. (F) Reporter assay in Jurkat T cells transfected with control or *Il5* promoter vectors and a PARP-14 expression vector. Average normalized luciferase values of triplicates are representative of two experiments.

The RNA pol II ChIP-seq dataset suggested that Th2 cytokines and *Gata3* are genes that require PARP-14 for normal expression. This was confirmed using qPCR (Figure 5A) and is consistent with our recent observations. PARP-14 functions as a transcriptional switch to regulate STAT6 dependent transcription, binding DNA in the absence of IL-4 to act as a transcriptional repressor [25]. The nature of the DNA element that PARP-14 binds to is not known. To determine the identity of a PARP-14-binding DNA element we performed de novo motif analysis using HOMER. The DNA sequence corresponding to −1000 bp to 100 bp relative to the transcription start site for each gene in Pool 2 was analyzed using HOMER, with genes not dependent on PARP-14 as background. The de novo analysis identified 28 potential motifs that were enriched in genes in Pool 2 (data not shown). Among these were a GATA3 motif enriched 4-fold over background (5.7% of genes) and IRF motifs enriched 3-fold over background (15.8% of genes). Motifs that did not correspond to known binding sites and showed an enrichment of 3.5 or greater in the target genes as compared to background genes were deemed to be the putative DNA binding motifs for PARP-14. These analyses resulted in six putative motifs. Four motifs had repeat sequences and were considered to be false positives.

We next searched for the presence of the remaining two motifs in the genes that we had validated as positively regulated by PARP-14. Thus, the *Il4*, *Il5*, *Il13*, *Il21*, *Gata3* and *Stat6* loci (±10 kb of the gene) were scanned for the presence of these motifs allowing for a mismatch in the 10-base long motif less than or equal to 1, and in the 12-base long motif less than or equal to 2. Motif 1 - CACTGAGTGGAG and Motif 3– TCCAAGGATC were found in the promoters of *Il5* and *Il4*, respectively (Figure 5B and C).

In order to validate motifs 1 and 3 as legitimate binding sites for PARP-14, we performed DNA Affinity Pull-down Assays (DAPA). Oligonucleotides corresponding to the motifs found in the promoter regions of *Il4* and *Il5* were synthesized, along with scrambled controls (Figure 3B), to perform DAPA with extracts from PARP-14 transfected cells. PARP-14 showed specific binding to DNA elements corresponding to regions within the *Il4* and *Il5* promoter, containing motif 3 and 1 respectively (Figure 5D and E). In contrast, reduced PARP-14 binding to these elements was observed when motif 3 and 1 were scrambled (Figure 5D and E). To demonstrate a functional role for PAPR-14 in regulating these genes, we performed a reporter assay where a PARP-14 expressing plasmid was co-transfected with luciferase vectors containing the *Il5* or an irrelevant promoter. Consistent with the demonstrated role for PARP-14 in repressing basal (uninduced) gene expression [25], we observed PARP-14 repressed reporter expression from the *Il5* promoter (Figure 5F). Thus, we have identified putative PARP-14 binding elements in genes that are regulated by PARP-14.

## Discussion

In this study we have identified on a genomic scale, the genes in Th2 cells that are regulated by PARP-14, a cofactor that functions with STAT6. Our approach used ChIP-Seq analysis with an antibody directed against the active form of RNA polymerase II to identify genes that were actively being transcribed. This approach captures active transcription of a gene precisely and is not affected by the half-life of transcripts that impacts microarray analysis. Using this approach we were able to identify 2,744 genes whose expression in Th2 cells was dependent on the expression of PARP-14. A majority of the genes were positively regulated by PARP-14. However for 430 genes the expression was higher in samples lacking PARP-14 expression. We did observe that a number of microRNAs were positively regulated by PARP-14, and speculate that PARP-14 may negatively regulate expression of some of the 430 genes through a mechanism involving microRNAs. Further analysis and experimentation will be required to confirm this speculation. From our analysis we were also able to discern the genes whose expression was dependent on the ART activity of PARP-14 and that were independent of ART activity. We found that 1,647 (Pool 4) genes were regulated by PARP-14 but not by ART activity, genes that included *Il4ra*, *Il7r* and *Il10*. Our data indicated that 1,097 genes required ART activity and 756 of these genes (Pool 5) required the ART activity of only PARP-14. This subset of genes included *Il2*, *Il21*, *Il12rb1* and *Il18rap*. We also inferred that 341 genes (Pool 6) were dependent on the ART activity of PARP-14 and/or other PARP enzymes (Figure 1A and Table S7), suggesting that multiple PARP enzymes may collaborate to regulate gene expression. All of the Th2 cytokine genes including, *Il4, Il5* and *Il13* belonged to this Pool indicating that these genes were predominantly regulated by the both PARP-14 and ART activity. This observation was consistent with Datta et al who have elegantly showed that IL-5 was regulated by PARP-1 and its activity [39]. Thus, with these analyses we have been able to segregate genes into categories that have a specific requirement for PARP-14 dependent or independent of PARP-14 ART activity, and those that have a more complex requirement for PARP-14 and ART activity which may include the ART activity of PARP-14 or other PARP family enzymes. These data also suggest that in addition to PARP-14 there may be other PARP enzymes that regulate gene expression in Th2 cells. Our previous work has demonstrated that for the *Fcer2a* and *Ie* genes, the ART activity of PARP-14 plays an important role in its function of enhancing STAT6 dependent transcription [25]. Here we find that for some of the Th2 genes the ART activity of PARP-14 may not be required. This indicates that PARP-14 may also regulate transcription using additional mechanisms independent of ART activity and unique from what we have described earlier.

The DAVID analysis we performed indicated that PARP-14 regulates genes that participate in a number of cellular pathways, including the ribosomal machinery, T cell receptor signaling, ubiquitin mediated proteolysis, cell cycle and MAPK signaling pathway. We found that most of the genes within these pathways were positively regulated by PARP-14 as the gene expression was higher in *Parp14*+/+ as compared to *Parp14*−/−. These data indicate that PARP-14 may be involved in general cellular pathways not unique to Th2 cells. It is unclear whether the genes within these pathways are directly transcriptionally regulated by PARP-14, or through an alternate mechanism. It will be important to determine if these pathways are similarly regulated in other T helper cell subtypes or whether PARP-14 regulates these pathways only in Th2 cells. From the DAVID analysis we identified that the gene expression of almost 59 ribosomal genes was dependent on the ART activity of only PARP-14. None of these ribosomal genes were regulated by STAT6 indicating that PARP-14 may function independent of STAT6 in Th2 cells. This is a very interesting finding, and it will be important to experimentally determine if PARP-14 and its enzyme activity regulate protein synthesis and again if this is specific to Th2 cells, although such studies are beyond the scope of the present study.

Consistent with the role of PARP-14 in STAT6 dependent transcription we found a number of genes under the control of the JAK-STAT pathway to be dependent on PARP-14. These included some of the SOCS genes and we verified experimentally the dependence of PARP-14 on the expression of these genes (Figure 3C). We found that both SOCS1 and 3 were negatively regulated by PARP-14. SOCS1 is known to block Th2 differentiation by inhibiting the IL-4/STAT6 pathway [40]. Thus, we speculate that PARP-14 may aid Th2 differentiation by inhibiting the expression of SOCS1, such that SOCS1 is unable to inhibit the IL-4/STAT6 axis. Consistent with the positive role of STAT6 in the expression of Th2 cytokines we found that PARP-14 regulated the expression of Th2 cytokines as indicated above. It is also well established that in Th2 cells the expression of the Th1 specific transcription factors are inhibited [41]. The expression of IRF-1 is enriched in Th1 cells and plays an important role in Th1 but not Th2 differentiation [33], [35]. We observed that in *Parp14*−/− Th2 samples, the expression of *Irf1* was higher suggesting that PARP-14 may promote Th2 differentiation by inhibiting the expression of transcription factors that promote the opposing Th1 differentiation. We compared the list of PARP-14-dependent genes with genes that are enriched in Th2 cells from two separate microarray experiments [42], [43] and found that 2–4% of the PARP-14 regulated genes could be considered as enriched in Th2 cells. From our ChIP-Seq analysis we also observed that PARP-14 and its enzyme activity regulated the expression of *Il21*, and this was verified experimentally in Th2 cells (Figure 5A). IL-21 is produced by most T helper subsets and plays an important role in immunoglobulin secretion [44]. More recently it has been determined that IL-21 promotes Th17 and T follicular helper (Tfh) cell development and function [44], [45], [46]. Thus, we speculate that besides playing a role in Th2 differentiation PARP-14 may also play a role in other T helper cell subsets including Th17 and Tfh. PARP-14 promotes Th9 differentiation, indicating that PARP-14 is not restricted to the Th2 subset [26].

Our analysis has identified two putative PARP-14 binding sites Motif 1– CACTGAGTGGAG and Motif 3– TCCAAGGATC (Figure 5). Both of these motifs were also found in the *Fcer2a*, *Gata3* and *Ie* genes –genes directly regulated by PARP-14 in a STAT6-depedent manner [24], [25], [47]. Furthermore, we have determined here that these two motifs are also found on the *Il4, Il5* and *Il13* loci. We have experimentally validated by DAPA that PARP-14 binds to the region of *Il4* and *Il5* that contains these motifs (Figure 5). Previously a number of DNase I hypersensitivity (HS) sites have been identified within the Th2 cytokine locus and have been shown to play a critical role in Th2 cytokine expression (reviewed in [41]). Indeed, we found Motif 1 to be located approximately 1.2 kb, and Motif 3 to be located approximately 350 base pairs away from the DNase I HS I in the IL-4 promoter region. We also found the CACTGAGTGGAG motif near HSS 1 and 2 in the IL-4/IL-13 intergenic region which when deleted, reduces IL-4 and IL-13 levels [48]. This motif was also found near the HS V_A_ site of the IL-4 gene that is also known as the IL-4 3′ enhancer [49]. It is known that STAT6 binds to both the HS I and V_A_ regions of the IL-4 promoter [49]. Taken together, these data suggest that PARP-14 may be regulating the IL-4 locus directly through a STAT6-dependent mechanism. However, HOMER analysis of all PARP-14-dependent genes did not identify STAT consensus sites, suggesting that PARP14 is preferentially associated with STAT targets that lack a consensus STAT site, that STAT sites are largely at a greater distance from the promoter in most target genes, or that STATs represent only one of the many functional partners of PARP-14. The enrichment of binding sites for GATA and IRF factors, both of which contribute to Th2 development, are consistent with the latter explanation.

## Conclusions

We have identified on a genomic scale the genes in Th2 cells that are regulated by PARP-14 and ART activity. Our data indicate that the gene expression dependent on PARP-14 can be either modulated by, or independent of ART activity. Importantly, our data demonstrate that PARP-14 functions with STAT6 to regulate gene expression of many hallmark genes in Th2 cells. We have also identified putative DNA binding sites for PARP-14 and find these sites in the genes that are regulated by PARP-14. Together, these data provide important new insight into the biological functions of PARP-14.

## Supporting Information

Table S1 **List of genes that are actively transcribed as determined by RNA Polymerase II ChIP-Seq in Th2 cells.** Related to Figure 1.
(XLS)

Table S2 **Pool 1– Filtered list of genes that are significantly transcribed as determined by RNA Polymerase II ChIP-Seq in Th2 cells.** Related to Figure 1.
(XLS)

Table S3 **Pool 2 - List of genes that show a dependence on PARP-14 for their transcription.** Related to Figure 1.
(XLS)

Table S4 **Pool 3 - List of genes that show a dependence on PARP catalytic activity for their transcription.** Related to Figure 1.
(XLS)

Table S5 **Pool 4 - List of genes that are independent on PARP enzymatic activity.** Related to Figure 1.
(XLS)

Table S6 **Pool 5 - List of genes that are dependent on only PARP-14 enzymatic activity.** Related to Figure 1.
(XLS)

Table S7 **Pool 6 - List of genes that are dependent on enzymatic activity of PARP-14 and other PARPs.** Related to Figure 1.
(XLS)

Table S8 **Tab 1** **.** List of genes positively regulated by PARP enzymatic activity of other PARPs but are negatively regulated by PARP-14. Table S8 Tab 2. List of genes positively regulated by PARP-14 enzymatic activity. Table S8 Tab 3. List of genes positively regulated by PARP-14 but independent of its enzymatic activity. Table S8 Tab 4. List of genes negatively regulated by PARP-14 but not by its enzymatic activity. Table S8 Tab 5. List of genes negatively regulated by PARP-14 enzymatic activity. Table S8 Tab 6. List of genes negatively regulated by PARP activity of other PARPs but positively regulated by PARP-14. Related to Figure 1.
(XLS)

Table S9 **Tab 1** **.** List of genes positively regulated by STAT6 and PARP-14 and that bind STAT6. Table S9 Tab 2. List of genes positively regulated by PARP-14 and independent of STAT6. Table S9 Tab 3. List of genes that bind STAT6 but are not positively regulated by STAT6 and PARP-14. Table S9 Tab 4. List of genes positively regulated by STAT6, and that do not bind STAT6, and are not positively regulated by PARP-14. Table S9 Tab 5. List of genes positively regulated by PARP-14, and bind STAT6 but are not positively regulated by STAT6. Table S9 Tab 6. List of genes positively regulated by STAT6 and PARP-14 but do not bind STAT6. Table S9 Tab 7. List of genes positively regulated by STAT6 and bind STAT6 but are not positively regulated by PARP-14. Table S9 Tab 8. List of genes negatively regulated by STAT6 and PARP-14. Table S9 Tab 9. List of genes negatively regulated by PARP-14 but not by STAT6. Table S9 Tab 10. List of genes negatively regulated by STAT6 but not by PARP-14. Related to Figure 5.
(XLS)
